# Supplementary material for: Australian vegetated coastal ecosystems as global hotspots for climate change mitigation
Source: Nat Commun. 2019 Oct 2;10:4313. doi: 10.1038/s41467-019-12176-8 (PMC6773740; doi:10.1038/s41467-019-12176-8)
Supplement: Supplementary file 2 — Supplementary Information [file 41467_2019_12176_MOESM2_ESM.pdf]

Australian vegetated coastal ecosystems as global hotspots for climate change mitigation

Serrano et al.

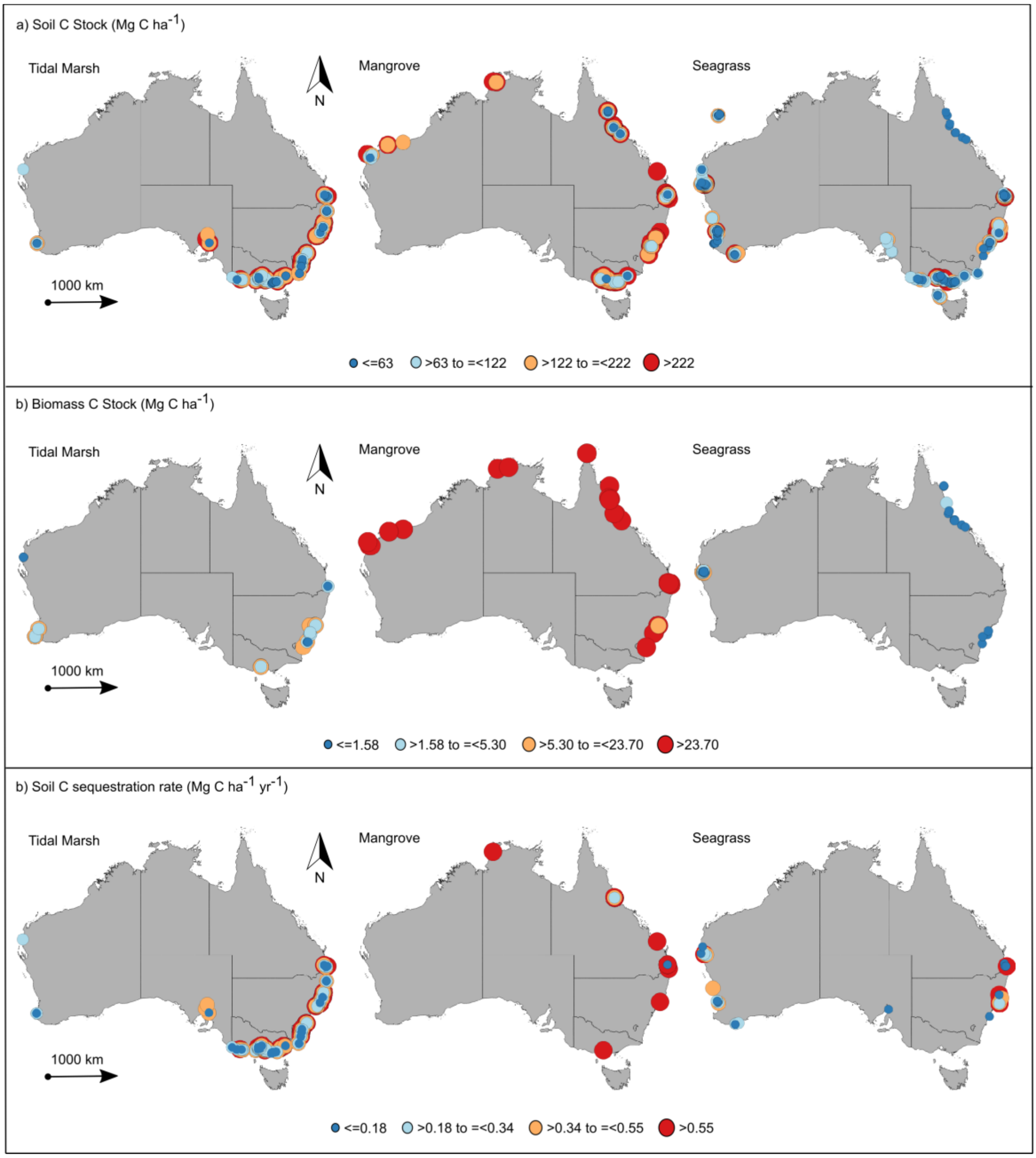

Supplementary Figure 1. Direct measurements of organic carbon (C) storage in vegetated coastal ecosystems (i.e. tidal marshes, mangroves and seagrasses) across Australia. **a)** Soil C storage (Mg C ha^{-1}) in the top meter. **b)** Living aboveground biomass C stock (Mg C ha^{-1}). **c)** Soil C sequestration rates (Mg C ha^{-1} yr^{-1}). The size and colour of dots show the magnitude of C storage: C stocks in living aboveground biomass and the top meter of soil ranging from <1.58 to >222 Mg C ha^{-1}, and soil C sequestration rates ranging from <0.18 to >0.55 Mg C ha^{-1} yr^{-1}. The four ranges of data (indicated by different

colours) are based on the lower quartile, median quartile, and upper quartile. Source data are provided as a Source Data file.

Supplementary Table 1. General Linear Models. Living aboveground biomass organic carbon (C) stock, soil C stock (in the top meter) and soil C sequestration rates in response to climate region and ecosystem type (fixed effects), and interaction between climate region and ecosystem type. Climate region: arid, semi-arid, temperate, subtropical and tropical. Ecosystem: tidal marsh, mangrove, seagrass. Significant interactions from post-hoc HSD tests are indicated with numbers in superscript ($P < 0.05$).

Variable	Factor	df	Mean Square	F	P
Aboveground biomass C stock	[1]Climate region	4	53,417,196	2.94	**0.023**
(Mg C ha^{-1})	[2]Ecosystem	2	1,047,413,681	57.65	**<0.001**
	[3]Climate region * Ecosystem	3	68,069,883	3.75	**0.013**
Soil C stock	[4]Climate region	4	40,784	3.08	**0.016**
(Mg C ha^{-1})	[5]Ecosystem	2	532,031	40.17	**<0.001**
	[6]Climate region * Ecosystem	7	93,491	7.06	**<0.001**
Soil C sequestration rates	[4]Climate region	4	4,395	3.45	**<0.01**
(Mg C ha^{-1})	[2]Ecosystem	2	26,156	20.55	**<0.001**
	[7]Climate region * Ecosystem	5	3,012	2.37	**0.039**

[1]Subtropical = Tropical > Temperate = Arid

[2]Mangrove > Tidal marsh = Seagrass

[3]Tidal marsh: Temperate > Semi-arid = Subtropical

[3]Mangrove: Tropical > Temperate

[3]Seagrass: Arid > Temperate = Tropical

[4]Tropical > Arid = Semi-arid = Temperate = Subtropical

[5]Mangrove > Tidal marsh > Seagrass

[6]Seagrass: Arid > Subtropical

[6]Mangrove: Subtropical > Temperate = Tropical = Arid = Semi-arid

[7]Mangrove: Tropical > Subtropical

Supplementary Table 2. Organic carbon (C) storage within Australian climate regions. Estimates of area (in ha) and C stocks in living aboveground biomass and soils (in Mg C ha^{-1}), and C sequestration rates (Mg C ha^{-1} yr^{-1}) in vegetated coastal ecosystems (i.e. tidal marshes, mangrove forests and seagrass meadows) within Australian climate regions. * Estimates based on the nearest climate region when data was not available. Mean and median ± SD.

Region	Ecosystem	Variable	Climate region<br>Arid	Semi-arid	Temperate	Subtropical	Tropical
AUSTRALIA	Tidal marsh	Area (ha)	51,392	327,609	90,087	118,475	945,360
		Stock - Aboveground biomass (Tg C)	0.05*	0.33	0.75	0.16	1.3*
		Stock - Soil (Tg C)	11.3	44.4	15.5	18.1	145*
		Sequestration rates (Tg C yr^{-1})	0.03	0.10	0.04	0.04	0.33*
	Mangrove	Area (ha)	23,412	100,180	13,706	151,002	762,577
		Stock - Aboveground biomass (Tg C)	2.8	11.1*	0.97	15.3	128
		Stock - Soil (Tg C)	3.3	15.1	3.4	55.3	180
		Sequestration rates (Tg C yr^{-1})	0.02*	0.08*	0.02	0.13	1.2
	Seagrass	Area (ha)	2,105,141	888,069	774,155	4,866,351	4,138,299
		Stock - Aboveground biomass (Tg C)	5.4	2.3	0.21	12.4	1.9
		Stock - Soil (Tg C)	273	108*	78.2	439*	153
		Sequestration rates (Tg C yr^{-1})	0.80	0.10	0.33	1.3	1.1*

Supplementary Table 3. Organic carbon (C) storage per unit area within climate regions. C stocks in living aboveground biomass and soils (in Mg C ha^{-1}), and sequestration rates (Mg C ha^{-1} yr^{-1}) in vegetated coastal ecosystems (i.e. tidal marshes, mangrove forests and seagrass meadows) within Australian climate regions. Mean and median ± SD.

Region	Ecosystem	Variable	Climate region																			
			Arid				Semi-arid				Temperate				Subtropical				Tropical			
			N	Mean	Median	SD	N	Mean	Median	SD	N	Mean	Median	SD	N	Mean	Median	SD	N	Mean	Median	SD
AUSTRALIA	Tidal marsh	Stock - Aboveground biomass (Mg C ha^{-1})	n/a	n/a	n/a	n/a	3	1.0	1.0	0.09	46	8.3	7.2	6.0	3	1.4	1.5	0.4	n/a	n/a	n/a	n/a
		Stock - Soil (Mg C ha^{-1} in 1 m-thick)	2	221	221	25	4	136	137	89	220	173	141	121	66	153	135	147	n/a	n/a	n/a	n/a
		Sequestration rates (Mg C ha^{-1} yr^{-1})	2	0.51	0.51	0.06	4	0.31	0.31	0.20	220	0.40	0.32	0.28	66	0.35	0.31	0.34	n/a	n/a	n/a	n/a
	Mangrove	Stock - Aboveground biomass (Mg C ha^{-1})	8	121	75	95	n/a	n/a	n/a	n/a	9	70	86	41	5	101	78	64	15	167	159	101
		Stock - Soil (Mg C ha^{-1} in 1 m-thick)	7	141	124	64	10	151	125	95	106	247	234	116	34	366	311	250	105	236	250	141
		Sequestration rates (Mg C ha^{-1} yr^{-1})	n/a	n/a	n/a	n/a	n/a	n/a	n/a	n/a	2	1.31	1.31	0.13	9	0.85	0.84	0.41	13	1.5	1.00	1.09
	Seagrass	Stock - Aboveground biomass (Mg C ha^{-1})	36	2.5	2.1	2.1	n/a	n/a	n/a	n/a	4	0.27	0.23	0.21	n/a	n/a	n/a	n/a	12	0.46	0.21	0.57

Stock - Soil (Mg C ha^{-1} in 1 m-thick)	164	130	125	75	15	121	98	109	241	113	83	95	120	90	65	83	9	37	48	22
Sequestration rates. (Mg C ha^{-1} yr^{-1})	8	0.38	0.27	0.31	1	0.11	n/a	n/a	12	0.50	0.30	0.44	15	0.26	0.20	0.19	n/a	n/a	n/a	n/a

Supplementary Table 4. Organic carbon (C) storage within Australian administrative jurisdictions. Estimates of area (in ha) and C stocks in living aboveground biomass and soils (in Mg C ha^{-1}), and C sequestration rates (Mg C ha^{-1} yr^{-1}) in vegetated coastal ecosystems (i.e. tidal marshes, mangrove forests and seagrass meadows) within Australian jurisdictions. Mean and median ± SD.

			State						
Region	Ecosystem	Variable	NT	QLD	NSW	VIC	TAS	SA	WA
AUSTRALIA	Tidal marsh	Area (ha)	428,038	601,808	15,399	49,655	12,719	48,096	377,210
		Stock - Aboveground biomass (Tg C)	0.57	0.78	0.09	0.41	0.11	0.16	0.45
		Stock - Soil (Tg C)	65.0	90.2	2.6	8.6	2.2	7.8	57.6
		Sequestration rates (Tg C yr^{-1})	0.15	0.21	0.0003	0.02	0.01	0.02	0.13
	Mangrove	Area (ha)	384,679	406,097	12,726	5,746	0	29,297	212,333
		Stock - Aboveground biomass (Tg C)	63.5	57.6	1.0	0.40	n/a	3.3	32.0
		Stock - Soil (Tg C)	89.4	114	3.7	1.4	n/a	4.4	44.3
		Sequestration rates (Tg C yr^{-1})	0.58	0.51	0.01	0.01	n/a	0.02	0.28
	Seagrass	Area (ha)	666,629	8,275,771	20,881	262,234	84,500	962,000	2,500,000
		Stock - Aboveground biomass (Tg C)	0.34	13.8	0.02	0.07	0.02	2.4	5.5
		Stock - Soil (Tg C)	26.3	562	2.2	29.7	9.6	119	311
		Sequestration rates (Tg C yr^{-1})	0.17	2.1	0.01	0.13	0.04	0.19	0.94
